# Thrombolytic and anticoagulant therapy for acute submassive pulmonary embolism

**DOI:** 10.3892/etm.2013.1355

**Published:** 2013-10-22

**Authors:** JIANWEN FEI, YAN TANG, JINXIANG WU, LIJUN KANG, JIPING ZHAO, HONG DAI, WENXIANG BI, JUNFEI WANG, FEN LIU, WEN LIU, MENG YANG, LIANG DONG

**Affiliations:** 1 Department of Pulmonary Medicine, Qilu Hospital of Shandong University, Jinan, Shandong 250012, P.R. China; 2 Department of Pulmonary Medicine, Yantai Shan Hospital, Yantai, Shandong 264001, P.R. China; 3 Institute of Biochemistry and Molecular Biology, School of Medicine, Shandong University, Jinan, Shandong 250012, P.R. China

**Keywords:** submassive, pulmonary embolism, anticoagulation, thrombolysis

## Abstract

This study aimed to compare the efficacy and safety of thrombolytic and anticoagulant therapy for acute submassive pulmonary embolism (PE). A retrospective evaluation was performed on 25 consecutive inpatients with acute submassive PE treated by thrombolytic therapy and 25 earlier consecutive inpatients with acute submassive PE treated by anticoagulant therapy. No statistically significant difference in clinical curative effect was identified between the thrombolysis and anticoagulation groups (P>0.05). Following 24 h of therapy, the improvement rates of dyspnea and revascularization in the thrombolysis group achieved statistical significance compared with those of the anticoagulation group (P<0.01 for each). The PO_2_ level of the thrombolysis group (81.18±5.66 mmHg) was notably higher than that of the anticoagulation group and the difference was statistically significant (P<0.01). The pulmonary arterial pressures of the thrombolysis group (51.21±6.86 mmHg) were significantly lower than those of the anticoagulation group (60.64±5.17 mmHg) (P<0.01). Furthermore, the difference between the hemorrhage rates of the two groups was statistically significant (P<0.05). Thrombolysis was shown to rapidly relieve dyspnea, reduce pulmonary arterial pressure and revascularize the embolized blood vessels. However, the hemorrhage rate of the thrombolysis group was higher than that of the anticoagulation group. The overall efficacies and fatality rates of the thrombolysis and anticoagulation groups were similar.

## Introduction

Pulmonary embolism (PE) is a clinical and pathophysiological syndrome caused by endogenous or exogenous embolysis infarcting the pulmonary artery or its branches and inducing disturbances in the pulmonary circulation. The incidence and fatality rates of PE are high. The fatality rate of untreated PE is 30%; however, early diagnosis and active treatment may reduce the fatality rate by between 3 and 10% (1–3).

PE is classically subdivided into massive, submassive and nonmassive stratifications. Massive PE occurs with hemodynamic instability [systolic blood pressure (BP) <90 mmHg]. Nonmassive PE is defined as normotensive PE without signs of right ventricular dysfunction (RVD) and myocardial damage, while submassive PE is defined as normotensive PE with signs of RVD and/or myocardial damage. The short-term mortality rate of submassive PE ranges from 3 to 15% (4,5). For nonmassive PE, the use of anticoagulant therapy is routine. However, the appropriate therapy for submassive PE remains controversial (6).

While anticoagulation treatment does not directly dissolve the clot, it is the basic treatment for PE, as it halts the propagation of the thrombus, leaving the existing thrombus to the natural fibrinolytic system. It is necessary for anticoagulation treatment to be performed immediately, at the first instance of clinical suspicion of PE (7). Compared with thrombolytic therapy, anticoagulant therapy is mild and may feature hemorrhagic complications. Gould *et al*(8) revealed that the incidence of major bleeding from anticoagulant therapy was 1.9%, with a fatal hemorrhage rate of 0.2%. Thrombolytic therapy was first introduced to treat PE in the 1960s and has continued to provide marked angiographic improvements (9). Thrombolytic therapy has since been uninterruptedly administered to patients with acute PE, resulting in significant treatment efficiency rates (10–13). Thus, thrombolytic therapy is a widely accepted standard treatment for patients with hemodynamic instability (5). However, thrombolytic therapy presents several disadvantages. For instance, thrombolytic therapy has increased bleeding risks compared with anticoagulant therapy (14). Thrombolytic therapy is able to directly dissolve clots to accelerate the resolution of PE, which may appear to be more effective compared with the use of anticoagulants. However, it is necessary for physicians to assess the thrombolytic benefits against the significantly increased hemorrhagic risks (14,15). To date, the clinical outcomes of thrombolytic therapy remain incompletely understood, which further increases the controversy of thrombolytic therapy in submassive PE. The present study aimed to evaluate the treatment of acute submassive PE with standard thrombolytic or anticoagulant therapy and compare the efficacies and safeties of the two treatment procedures.

## Materials and methods

### Patients and methods

A retrospective study was undertaken, in which a total of 50 consecutive inpatients with acute submassive PE from January 2005 to August 2011 were selected. The inclusion criteria were as follows: age ≤75 years and disease period <1 week. The exclusion criteria included patients with chronic thromboembolic pulmonary hypertension without recent new-onset acute pulmonary embolism, patients with active visceral bleeding or spontaneous intracranial hemorrhage or those with a history of cardiopulmonary resuscitation, a blood platelet count of <100×10^9^/l, patients with minimally controlled severe hypertension, liver/kidney insufficiency or diabetic hemorrhagic retinopathy and pregnant patients. The selection yielded 34 male and 16 female patients, ranging in age from 39 to 72 years (average age, 57.22±8.97 years). The present study was conducted in accordance with the Declaration of Helsinki and with approval from the Ethics Committee of Qilu Hospital of Shandong University (Jinan, China). Written informed consent was obtained from all participants. Following treatment and analysis, the two groups did not show statistically significant variances in clinical data, such as age, gender, embolism location and concomitant diseases (P>0.05). For the thrombolysis group, 20,000 U/kg urokinase was added to 100 ml physiological saline, which was infused using an infusion pump for 2 h. The thrombolysis treatment was a single 2-h infusion only. Subsequent to the completion of the thrombolytic therapy, the activated partial thromboplastin time (APTT) of the patients was checked every 4 h. Following recovery to less than two-fold the basic control value, fraxiparine (0.1 ml/10 kg) was infused by subcutaneous injection. For the anticoagulation group, fraxiparine (0.1 ml/10 kg) was infused by subcutaneous injection every 12 h, for 14 days. Warfarin was subsequently administered as an oral anticoagulant therapy for ≥3 months.

### Observation items and indices

Symptoms and signs, blood gas and D-dimer levels, echocardiography test results and computed tomography pulmonary angiography (CTPA) findings were monitored prior to and following each instance of thrombolytic or anticoagulant therapy.

### Adverse reaction observation

The incidences of hemorrhage, dermatorrhagia, visceral bleeding and intracranial hemorrhage, as well as the incidence of heparin-induced thrombocytopenia (HIT), were observed.

### Main index of performance assessment

The performances of the two therapies were assessed using calculations of total effectiveness (curative rate + excellent rate + effective rate), hemorrhage and mortality rates.

### Standards for judging the clinical curative effect

Clinical curative effect was classified with the following terms: i) ‘Cure’ meant that the dyspnea had predominantly disappeared, the chest pain was totally relieved and that the patient was cured or basically cured according to the imaging results; ii) ‘excellent’ meant that it was the dyspnea that had markedly disappeared, the chest pain was markedly relieved and imaging showed effectiveness; iii) ‘efficiency’ meant that the dyspnea was ameliorated, the chest pain was partially relieved and imaging showed effectiveness; iv) ‘inefficiency’ meant that the dyspnea and chest pain were not ameliorated and that imaging showed no effectiveness; v) ‘aggravation’ meant that symptoms, such as dyspnea and chest pain, were worse and that imaging showed a deterioration; and vi) mortality.

### Statistical analysis

Statistical analysis was performed using the SPSS 10.0 statistical software package (SPSS, Inc., Chicago, IL, USA). Data were compared using the Student’s t-test and the U-test. Measurement data are presented as the mean ± SD P<0.05 was considered to indicate a statistically significant result.

## Results

Of the 50 enrolled patients, 25 underwent thrombolysis and 25 received anticoagulant therapy. The two groups had a similar demographic baseline. After 24 h, the thrombolytic therapy was shown to significantly improve the rate of dyspnea compared with the anticoagulant therapy (P<0.01); however, no statistical differences were observed between the two treatments after 7 and 14 days and 1 month (Table I). Twenty-four hours after treatment, the chest pain was ameliorated in 28.6% of the patients in the thrombolysis group compared with 20.0% in the anticoagulation group (P>0.05). At 7 days after treatment initiation, 71.4 and 80.0% of the patients in the thrombolysis and anticoagulation groups, respectively, showed improvements with regard to chest pain (P>0.05), while at 14 days, improvements were shown in 85.7 and 80.0% of the patients, respectively (P>0.01). None of the patients had any chest pain after 1 month (Table II). After 24 h, the partial pressure of oxygen (PO_2_) of the thrombolysis group (81.2± 5.7 mmHg) was markedly higher than that of the anticoagulation group (74.9±8.4 mmHg) (P<0.01); however, no statistically significant differences were observed between the groups after 7 and 14 days and 1 month (Table III). Twenty-four hours after thrombolytic therapy, the D-dimer levels of the thrombolysis group increased. Furthermore, the difference between the groups was statically significant (t=2.205, P=0.037) at this time-point; however, no significant differences in D-dimer levels were observed after 7 and 14 days and 1 month (Table IV). No statistically significant differences were observed between the two groups with regard to the partial pressure of carbon dioxide (PCO_2_; Table V). Twenty-four hours after therapy, the pulmonary arterial pressures of the thrombolysis group (51.2±6.9 mmHg) were significantly lower than those of the anticoagulation group (60.6±5.2 mmHg) (P<0.01); however, no significant differences were observed at 7 and 14 days and 1 month (Table VI). The revascularization rate 24 h after therapy in the thrombolysis group was significantly higher than that in the anticoagulation group (P<0.01); however, no statistically significant differences were observed between the groups at 14 days and 1 month (Table VII). The difference in hemorrhage rates between the two groups was statistically significant (P<0.05; Table VIII). It was observed that the type of hemorrhaging was subcutaneous hemorrhage in the two groups and the thrombolytic group had the higher hemorrhage rate. No statistical significance with regard to the difference in the clinical curative effects between the thrombolysis and anticoagulation groups was identified (P>0.05), and the fatality rates for the two groups were identical at 4% (P>0.05; Table IX).

## Discussion

PE remains a challenging condition to treat due to its high rate and mortality (3). Guidelines for the treatment of acute PE include those from the European Society of Cardiology, which recommends the use of thrombolytic therapy for massive PE and anticoagulant therapy for nonmassive PE (6). The 2008 American College of Chest Physicians Evidence-Based Clinical Practice Guidelines recommends that for submassive PE, thrombolytic therapy is an option for patients with a low risk of bleeding, with the administration of thrombolytics being dependent on the clinical assessment of PE severity, prognosis and bleeding risk (16). The use of thrombolytic agents in the treatment of submassive PE remains controversial.

Since the discovery of heparin in 1918, the anticoagulation method has been used for almost a hundred years to treat and prevent thromboembolic diseases. Anticoagulation is the basic treatment for PE since sufficient anticoagulant therapy is able to effectively prevent the propagation of the thrombus and dissolve the formed thrombus, via the fibrinolytic system of the body, thus reducing the number of mortalities from a relapse of pulmonary embolism (7). Sufficient anticoagulant therapy may avoid the relapse of pulmonary embolism and reduce fatality by 75% (17,18). However, anticoagulation treatment may result in several complications, such as hemorrhaging and HIT, with unfractionated or low-molecular weight heparin (7).

The use of thrombolytic therapy in the treatment of acute PE was initiated in the 1960s and was regarded as the ‘brave’ last therapeutic tool by the medical field at that time (9). Over the last few years, as highly qualitative thrombolytic drugs have been developed and new treatment protocols have been explored, the treatment protocol of thrombolysis has become increasingly advanced. Thrombolytic therapy directly dissolves the thrombi and more rapidly reverses hemodynamic instability than anticoagulant therapy (19). A study by Konstantinides *et al*(13) revealed that thrombolytic therapy significantly reduced mortality in submassive PE compared with heparin anticoagulation alone (13). No general consensus has been reached as to whether it is more effective for patients with acute submassive pulmonary thromboembolism to undertake thrombolytic or anticoagulant therapy. Certain questions with regard to thrombolytic therapy remain subjects of investigation, although thrombolytic therapy has adopted certain guidelines, such as ‘how to make patients get more benefits and less adventure’. The bleeding complications of thrombolytic therapy have been demonstrated to be notably higher than those of anticoagulant therapy; the overall major bleeding rate may reach up to 20% (20), while the risk of catastrophic intracranial hemorrhage is 1.9% (21). The present study was performed to compare the efficacy and safety of thrombolytic and anticoagulant therapies for acute submassive PE.

Efficacy and fatality rate are important indices used to determine the curative effect of medicine in cases of PE. In the present study, the total clinical efficacies of the two therapeutic protocols were 92 and 88%, respectively, and fatality rates were 4% for each, without statistical significances. With regard to the main clinical symptoms of PE (dyspnea and chest pain), all patients showed improvements subsequent to the therapies. Dyspnea began to improve between 30 min and 2 h after thrombolytic therapy, which was notably faster than with anticoagulant therapy. The difference between the two groups at 24 h after therapy was statistically significant (U=4.320, P<0.01). However, the two groups required 1–7 days for marked improvements and total remission, during which time the thrombolytic therapy dissolved or partially dissolved emboli to restore blood circulation to the lungs, improved the lung ventilation/perfusion ratio and relieved the symptoms of the patients. The chest pain of the patients began to improve 24 h after thrombolytic and anticoagulant therapy and was completely relieved at 7–14 days; no statistically significant differences were observed between the treatments. In the blood gas analysis, the majority of the patients showed different degrees of hypoxemia and hypocapnemia, indicating that changes in blood gas analysis have particular clinical value for the early diagnosis of PE. Improvements in the blood gas analysis of the thrombolysis group occurred earlier than in the anticoagulant group. The difference between the two groups 24 h after thrombolytic or anticoagulant therapy was statistically significant (t=2.980, P<0.01), indicating that blood gas analysis may be used as an indirect index for the early judgment of the efficacy of thrombolytic or anticoagulant therapy. The levels of D-dimers in the thrombolysis group increased 24 h after thrombolytic therapy and then decreased; by contrast, the D-dimer levels of the anticoagulation group showed a downward trend. The difference between the two datasets after 24 h was statistically significant (t=2.205, P=0.037). The D-dimer is a specific degradation product of cross filament protein under the fibrinolytic system (8,22). Thrombin forms markers hours subsequent to thrombolysis, resulting in a marked increase in the levels of prothrombin fragments (F1+2), thrombin-antithrombin III compounds and fibrinopeptides, indicating that D-dimers may reflect the dissolution rate of internal emboli. The increase in pulmonary arterial pressure in PE is the basis for a series of pathophysiological changes, such as impaired right ventricular function and shock. Mechanical blockage by thromboemboli in the pulmonary artery is the direct cause of increases in the pulmonary arterial pressure of patients with PE. Thrombolytic therapy may rapidly and partially release the mechanical blockage of pulmonary arteries, improve hemodynamics and reverse the impairment of right ventricular functions. Certain randomized controlled trials (RCTs) have demonstrated that 12 h subsequent to thrombolytic therapy with urokinase, there was a marked reduction in pulmonary arterial pressure, indicating that thrombolysis was able to rapidly reduce pulmonary arterial pressure (23). This observation is very beneficial for patients with acute submassive PE, particularly those under critical emergency care. The present study showed that the maximum systolic pulmonary arterial pressure of the thrombolysis group was notably decreased within the first day subsequent to thrombolytic therapy; this reduction was statistically significant compared with that of the anticoagulant group (t=−4.880, P<0.01). Changes in systolic pulmonary arterial pressure, determined by echocardiography, may be a good clinical index for determining the effectiveness of thrombolytic and anticoagulant therapy. Revascularization rate is also an important index by which to determine the treatment efficacy for PE. Based on the current study, the revascularization rate reached 22.9% in the first day subsequent to thrombolytic therapy, whereas only a 5.5% improvement was observed with anticoagulant therapy at this time-point; this difference was statistically significant (U=3.525, P<0.01).

The most significant complication of thrombolysis and anticoagulant therapy is bleeding. Bleeding may result in severe complications, such as intracranial hemorrhages, which are the primary cause of crippling or death in treatment; thus, bleeding is used as a main index to determine the efficacy of therapy. In the present study, the bleeding rate of the thrombolysis group was significantly higher compared with that of the anticoagulation group (U=2.085, P<0.05). Hemorrhea (including internal and intracranial hemorrhages) was not observed in either group. Bleeding was predominantly dermatorrhagia, particularly at the sites of paracentesis, which was consistent with other studies (24–26). Therefore, the present study demonstrated that thrombolytic and anticoagulant therapies were safe.

We suggest that patients in a critical condition with low risk of bleeding undergo thrombolytic therapy, while patients in a mild condition with high risk of bleeding undergo anticoagulant therapy. The risk of bleeding should be assessed sufficiently prior to the administration of medication. The identification of the patient’s blood group and preparation of blood for transfusion may be of benefit in certain cases. Prior to thrombolytic therapy, the use a peripheral vein trocar to draw blood for monitoring during the course of treatment it desirable, in order to reduce the incidence of blood vessel paracentesis and effectively reduce the bleeding rate. When necessary, deep veins should be punctured by intra-arterial injection, and pressure should be applied to the peripheral vein distal from the puncture site in case of local hematoma. Following future studies on large samples, we will focus close attention on the efficiency and risk of more time points between these two methods. Additionally, more questions regarding right heart insufficiency should be investigated.

## Figures and Tables

**Table I:** Improvement in dyspnea in the thrombolysis and anticoagulation groups.

	No. of patients with dyspnea (improvement rate, %)		
		
Time-point	Thrombolysis group	Anticoagulation group	P-value
Prior to treatment	25	25	
24 h after treatment	14 (44.0)	21 (16.0)	<0.01
7 days after treatment	2 (92.0)	3 (88.0)	>0.05
14 days after treatment	0 (100)	0 (100)	>0.05
1 month after treatment	0 (100)	0 (100)	>0.05

**Table II:** Chest pain improvements in the thrombolysis and anticoagulation groups.

	No. of patients with chest pain (improvement rate, %)		
		
Time-point	Thrombolysis group	Anticoagulation group	P-value
Prior to treatment	7	5	
24 h after treatment	5 (28.6)	4 (20.0)	>0.05
7 days after treatment	2 (71.4)	1 (80.0)	>0.05
14 days after treatment	1 (85.7)	1 (80.0)	>0.05
1 month after treatment	0 (100)	0 (100)	>0.05

**Table III:** Oxygen partial pressure (PO_2_) improvements in the thrombolysis and anticoagulation groups.

	PO_2_ (mmHg)		
		
Time-point	Thrombolysis group	Anticoagulation group	P-value
Prior to treatment	65.2±8.2	68.5±9.2	>0.05
24 h after treatment	81.2±5.7	74.9±8.4	<0.01
7 days after treatment	89.1±5.5	87.4±6.6	>0.05
14 days after treatment	92.1±5.4	91.4±5.0	>0.05
1 month after treatment	92.3±4.7	92.7±4.5	>0.05

**Table IV:** D-dimer improvements in the thrombolysis and anticoagulation groups.

	D-dimer (mg/l)		
		
Time-point	Thrombolysis group	Anticoagulation group	P-value
Prior to treatment	1.08±0.48	1.21±0.50	>0.05
24 h after treatment	1.27±0.53	0.95±0.47	<0.05
7 days after treatment	0.60±0.29	0.52±0.32	>0.05
14 days after treatment	0.39±0.16	0.36±0.19	>0.05
1 month after treatment	0.33±0.14	0.37±0.15	>0.05

**Table V:** Carbon dioxide partial pressure (PCO_2_) improvements in the thrombolysis and anticoagulation groups.

	PCO_2_ (mmHg)		
		
Time-point	Thrombolysis group	Anticoagulation group	P-value
Prior to treatment	32.2±4.9	33.7±5.3	>0.05
24 h after treatment	35.8±3.5	35.1±4.2	>0.05
7 days after treatment	39.3±3.8	38.9±3.5	>0.05
14 days after treatment	39.7±3.1	39.2±3.6	>0.05
1 month after treatment	40.3±3.8	40.8±3.4	>0.05

**Table VI:** Pulmonary arterial pressure improvements in the thrombolysis and anticoagulation groups.

	Pulmonary arterial pressure (mmHg)		
		
Time-point	Thrombolysis group	Anticoagulation group	P-value
Prior to treatment	64.8±6.2	63.6±6.2	>0.05
24 h after treatment	51.2±6.9	60.6±5.2	<0.01
14 days after treatment	35.2±7.0	36.7±8.0	>0.05
1 month after treatment	29.3±4.8	28.8±5.1	>0.05

**Table VII:** Revascularization rates in the thrombolysis and anticoagulation groups.

	Revascularization rate (%)		
		
Time-point	Thrombolysis group	Anticoagulation group	P-value
24 h after treatment	22.9	5.5	<0.01
14 days after treatment	54.7	52.1	>0.05
1 month after treatment	86.6	87.3	>0.05

**Table VIII:** Hemorrhagic complications of the two treatment protocols (n=25 per group).

Hemorrhagic complication	Thrombolysis group	Anticoagulation group	P-value
Subcutaneous hemorrhage (n)	3	1	
Gingival bleeding (n)	0	0	
Epistaxis (n)	0	0	
Hemoptysis (n)	0	0	
Microscopic hematuria (n)	0	0	
Total bleeding rate (%)	12	4	<0.05

**Table IX:** Clinical effect in the thrombolysis and anticoagulant groups (n=25 per group).

Clinical effect	Thrombolysis group	Anticoagulation group	P-value
Cure (n)	17	18	
Excellent (n)	3	2	
Efficiency (n)	3	2	
Inefficiency (n)	1	2	
Aggravation (n)	0	0	
Mortality (n)	1	1	
Total efficacy (%)	92	88	>0.05
